# Surface integrity assessment of endodontic instruments after repeated use: A microscopic analysis

**DOI:** 10.6026/973206300210817

**Published:** 2025-04-30

**Authors:** Faisal Naseem, Aishwarya Sinha Mathur, Keshav Kumar Manglam, Neha Verma, Vartika Singh, Shazia Siddiqui, Faizan Ahmad

**Affiliations:** 1 Department of Conservative Dentistry and Endodontics, Dental College, Azamgarh, Uttar Pradesh, India; 2 Department of Conservative Dentistry and Endodontics, Gautam Buddha Chikitsa Mahavidyalaya, Ras Bihari Bose Subharti University, Dehradun, Uttarakhand, India; 3 Department of Conservative Dentistry and Endodontics, Autonomous State Medical College, Bahraich, Uttar Pradesh, India; 4 Department of Prosthodontics, Government Dental College, Indore, Madhya Pradesh, India; 5 Department of Conservative Dentistry and Endodontics, Career Postgraduate Institute of Dental Sciences and Hospital, Lucknow, Uttar Pradesh, India; 6 Department of Conservative Dentistry and Endodontics, Career Postgraduate Institute of Dental Sciences and Hospital, Lucknow, Uttar Pradesh, India; 7 Frimley Park Hospital, Frimley, Camberley, UK, Europe

**Keywords:** Endodontics, pitting, root canal, rotary file, surface integrity

## Abstract

The surface condition of four Ni-Ti rotary file systems after using them to shape premolar root canals ten times are of interest. Laboratory tests through SEM showed substantial surface deterioration because pitting dramatically increased after ten procedures (p < 0.001). The Twisted File system remained in the best condition with the minimum amount of surface damage and second place went to ProTaper Next and V Taper 2H ranked after them and Mtwo showed the maximum wear. The surface wear observed in Mtwo exceeded that of all the other tested Ni-Ti rotary file systems. The assessment of file surfaces aids in minimizing clinical failures of instruments.

## Background:

The success of endodontic treatment is highly dependent upon root canal system preparation; consequently, the shaping of the canal should be accomplished in a continuously tapered form without disrupting its anatomical morphology to ensure that infected pulp tissue and microbial biofilm are removed in this manner, thus achieving the best kind of disinfection and obturation. Ni-Ti instruments have revolutionized endodontic practice. Ni-Ti instruments are much more predictable and efficient than stainless steel in preparing curved and narrow canals. They have excellent flexibility, increased torsional resistance and increased cutting efficiency; thus, reducing canal transportation and procedural errors to a great extent [1, 2]. These advantages are, however not free of some drawbacks of the instrument. Another common concern with clinical application is the possibility of sudden instrument separation. Unlike stainless steel files, which tend to show visible deformation before failure, Ni-Ti instruments often break without prior warning, making this a significant clinical challenge. Such a phenomenon might be due to cumulative stresses resulting in surface wear, micro-cracks and eventual fatigue failure. Such surface changes are critical in determining the performance and lifespan of Ni-Ti instruments and hence warrant sensitive integrity assessment following a cycle of utilization [3, 4]. Surface integrity in rotary instruments depends on a few factors. First, these relate to the physical properties of materials used and second, cross-section design of such instruments and also frequency and usage conditions. Generally, active cutting-edged instruments show higher efficiency during dentine removal but have surface wear due to higher friction forces. On the other hand, there is slower surface wear for a passive instrument and its radial land with a scraping instead of cutting effect, which may possibly take a much longer time [5, 6].

Aside from the orientation of the instrument, size and design, particularly the form of the cross-section, significantly affect the mechanical action of the instrument, which contributes to its deformation resistance. It has been reported that there is a possibility for higher torsional resistance for instruments with larger diameters or specific cross-sectional configurations, yet lower resistance to fatigue in clinical applications when reused [7, 8]. Rotary file systems have a good number of designs that differ with unique merits and limitations. The most widely used rotary systems include ProTaper Next, V Taper 2H, Twisted File and Mtwo. The characteristics of each of the rotary systems ensure differing cutting efficiency, flexibility and longevity. The cutting performance as well as flexibility of ProTaper Next system is based on a variable taper design. This means V Taper 2H has a continuous taper and is capable of having more resistance against cyclic fatigue, while Twisted File uses unique twisting that bestows it with flexibility and strength. Mtwo, however, uses an S-shaped cross-section that is designed for the removal of debris but with some compromise with resistance to fracture [9, 10]. Though various studies have investigated the efficiency in shaping and failure patterns of Ni-Ti instruments, little attention has been given to the progressive alterations of the surface that occur due to repeated usage. SEM would be a good tool for assessing these changes at high magnifications, which will give insight into the mechanisms of wear and failure. These changes should be understood to establish the safe limits of usage of rotary instruments and minimize the risk of instrument separation during clinical procedures [11]. Therefore, it is of ineterst to analyze the surface integrity of four Ni-Ti rotary systems, namely ProTaper Next, V Taper 2H, Twisted File and Mtwo, after repeated usage during root canal preparation.

## Materials and Methods:

## Sample collection and preparation:

Total 600 caries-free, single-rooted human premolars were selected for the study. The teeth were cleaned by hand scalers to remove debris and calculus and then rinsed with sodium hypochlorite to remove organic residues. After that, they were kept in distilled water. There were four groups representing a specific rotary system. They are (1) ProTaper Next (Dentsply), (2) V Taper 2H (SS White), (3) Twisted File (Sybron Endo) and (4) Mtwo (VDW).

## Inclusion and exclusion criteria:

Teeth with gross caries, restorations, severe attrition, erosion, fractures, open apices, calcified canal and significantly dilacerated were not included in the study. Each of these specimens was then categorized into four groups. In each of the groups, a total of 40 Ni-Ti instruments were used in the cleaning and shaping procedures.

## Experimental procedure:

Access cavities were prepared by the use of an Endo Access and Endo Z burs under continuous water cooling. The verification of canal patency and confirmation of working length was done by using a 10/02 K file. Preparation of canals up to the working length was done by using K files, *i.e.*, 10/02, 15/02 and 20/02 before entering the rotary systems.

## Grouping and instrumentation:

Group I: ProTaper Next (Dentsply). Instruments were placed in an X-Smart endomotor with Glyde File Prep lubricant with 10% carbamide peroxide and 15% EDTA. Irrigant used was 5% sodium hypochlorite followed by saline. Each instrument was inserted into five canals prior to sonic cleaning and SEM examination at x 500 magnification. Group II: V Taper 2H (SS White). Preparation protocol followed the same protocol as Group I. Files were set at 250 rpm with a torque of 65%. Instruments were utilized for five canals and SEM examined after debriding. Group III: Twisted File (Sybron Endo). Motor parameters are 500-600 rpm with a torque of 4-5. Every instrument prepared five canals before they were examined using SEM. Group IV: Mtwo (VDW). The instruments were utilized at 280 rpm with a torque of 1.1 Ncm in a crown-down sequence. After preparation, each file was ultrasonically cleaned and examined with SEM.

## Examination of surface changes:

Surfaces of the instruments were inspected before and after use through SEM (Zeiss Evo 18) at x 500 magnification. Photomicrographs were taken to check for pitting, deformation, or any changes. The observation was made by two observers independently without knowing the other's results for the unbiased interpretation. No instrument was autoclaved before performing the SEM study.

## Statistical analysis:

Statistical analysis was carried out to contrast usage cycles and surface wear. Descriptive statistics (means and standard deviations) quantified the pitting experienced by all groups. Intragroup variations within the four groups at varied usage intervals were revealed by one-way ANOVA, complemented by post-hoc Tukey's HSD tests to contrast between groups. Paired t-tests were applied to compare intragroup differences among usage intervals (5, 10 and 15 uses) to track the surface modification changes. All statistical analyses were carried out with SPSS software, Version 26.0 (IBM Corporation, USA) to make intergroup and intragroup comparisons of surface integrity.

## Results:

The mean pitting observed after five uses showed significant variability among the groups (Figure 1 - see PDF). Group IV (Mtwo files) exhibited the highest mean pitting value (12.20 ± 0.67), while Group III (Twisted File) demonstrated the least pitting (6.30 ± 0.75). Statistical comparisons revealed significant differences across all groups (p < 0.001). Based on surface integrity, the groups were ranked as follows: Group III > Group I > Group II > Group IV. The mean difference between Group I and Group II was -2.55 (p < 0.001), indicating lesser surface alterations in Group I compared to Group II (Table 1). Group I displayed significantly lower pitting compared to Group III and Group IV, with mean differences of -4.85 and -5.70, respectively (p < 0.001). Group II experienced more surface changes compared to Group III (-3.6, p < 0.001) and Group IV (-2.3, p < 0.001).

The progression of surface wear was evident after ten uses (Figure 2 - see PDF), with Group IV continuing to display the highest mean pitting (19.95 ± 0.86) and Group III showing the least (9.90 ± 0.81). All intergroup comparisons remained statistically significant (p < 0.001). The ranking of groups based on surface integrity remained unchanged: Group III > Group I > Group II > Group IV. After fifteen uses (Figure 3 - see PDF), surface degradation was most pronounced in Group IV (23.75 ± 1.06), while Group III maintained the lowest pitting levels (12.90 ± 0.61). Statistically significant differences (p < 0.001) were observed among all groups, with the ranking based on surface quality unchanged. Group I showed significantly less pitting than Group II (-2.35, p < 0.001), but higher pitting compared to Group III (4.9, p < 0.001) and Group IV (-5.95, p < 0.001) (Table 2). The difference between Group III and Group IV was particularly pronounced, with a mean difference of -10.85 (p < 0.001).

The trend of the surface alterations, showing pitting, was significant in all groups at various stages of the cycle (Table 3). Five and ten uses mean from Group I increased from 7.35 ± 0.85 to 13.05 ± 0.86, with a difference at a level of minus 5.70 (p < 0.001). This indicated the resultant of an enhanced trend of deterioration, indicating severe deterioration within these two intervals. From ten to fifteen uses, the mean pitting further increased from 13.05 ± 0.86 to 17.80 ± 0.89, having a mean difference of -4.75, with p values of less than 0.001. This signifies a continued progression of pitting in a meaningful and significant trend with increased usages of instruments. The greatest increase was reported when comparing five to fifteen uses. The mean pitting increased from 7.35 ± 0.85 to 17.80 ± 0.89, with a mean difference of -10.45 and a t-value of -27.26 (p < 0.001). This significant difference underlined the accumulating influence of repeated usage on the surface integrity of the instruments.

## Discussion:

One of the largest concerns of files still, related to endodontic treatment is their tendency towards file fracture. The properties related to structural behaviour and the geometrical design determine the resistance to the fracture of a NiTi endodontic instrument [12]. Despite these intrinsic qualities of the instruments, a new focus on recent evidence emphasizes technique and clinician handling as highly relevant to instrument durability and to file resistance towards fracture [13]. Traditionally, NiTi instruments were applied predominantly in continuous rotational motion. The paradigm shifted with the introduction of reciprocating motion for NiTi instruments by Yared *et al.* [14]. In later studies, reciprocating motion was found to increase the cyclic fatigue resistance of NiTi files in comparison to the traditional continuous rotation [15]. This innovation was built on by the new Twisted File system, developed with the use of the Elements Motor, incorporating both reciprocating and continuous rotational motions. The manufacturer reported that this "adaptive motion" decreased the mechanical stress imposed on the file and thus may contribute to increased safety and efficiency when preparing the root canal [16]. Flexibility is one important property that can influence the behavior of NiTi instruments. More flexible files tend to be of lesser torsional stiffness and tend to absorb deformation by allowing torsion applied to it. The performance is quite good in R-phase instruments, especially showing more angular deformation. Stress analyses are found to end up concluding that the internal structure is integral, particularly through structural surface integrity in maintaining integrity while torque is applied in torsional testing. As a result, instruments of R-phase show excellent resistance to failure induced by torsional overload, even in severe operating conditions. In the study, minimal surface degradation was revealed to be present as well with less pitting present in the Twisted File system followed by ProTaper Next V Taper 2H and Mtwo systems. Surface alterations were determined via scanning electron microscopy following cycles five, ten and fifteen which showed an increasing trend with the number of cycle. Manufacturing processes and phase transformations of NiTi alloys govern the mechanical properties of rotary files. Three phase transformations occur in NiTi alloys throughout its production; namely Austenite, Martensite and R-phase; [17]. For example, ProTaper Next instruments are produced from the M-Wire, a thermo-mechanically treated alloy of NiTi. In contrast to standard NiTi alloys, flexibility as well as file fatigue resistance improved through the M-Wire technology [18]. These instruments further offer higher values of Vickers hardness, therefore enhancing their sustainability [19]. Recent studies by Zinelis *et al.* are also in agreement, which showed that M-Wire-treated instruments exhibited higher hardness, compared to standard NiTi files [20]. The off-centered rectangular cross-section design of ProTaper Next files greatly determines its performance. It increases the cutting efficiency for files but incurs stress concentration that makes them vulnerable to fatigue. The use of lubricants has been highly recommended by manufacturers of rotary systems, especially EDTA, termed as RC Prep during the canal shaping procedure; this will reduce stress accumulation on instruments and walls. It has been proven that EDTA helps in reducing the accumulation of stress compared to saline during the process in curved canals. But if sodium hypochlorite is used as an irrigant, the possibility of pitting corrosion increases on the surfaces of the files [21].

Earlier researches have indicated that exposure to sodium hypochlorite has a degrading effect on NiTi instruments; such that it corrodes for longer periods greater than a few minutes. For instance, amounts of titanium were quantitatively observed after immersing Lightspeed NiTi files in 1% and 5% sodium hypochlorite solutions for 30 to 60 minutes [22]. Such exposure times also are not considered representative of a clinical condition in that instruments cannot remain in the oral cavity long enough for surface changes to result. To avoid even the possibility of misleading surface change under SEM, sodium hypochlorite was excluded. The lubricant and irrigant Glyde, normal saline were utilised instead so as to more nearly represent a clinic condition without risking corrosive artifact. Despite a lot of work on mechanical properties, Shen *et al.* [23] indicated that there is a lack of attention to the effects of precipitates and intermetallic compounds on NiTi properties. SEM observations of unused instruments in this work showed surface imperfections due to manufacturing processes. These defects are of concern since they act as initiation points for further degradation, as supported by Filho *et al.* [24]. Their research on K-files, NiTi instruments and Flexofiles documented manufacturing defects even before clinical use, which became worse with repeated use. Similarly, Parashos *et al.* [25] noted that clinical handling, particularly the operator's skill level, was a major factor in the formation of defects. Their results highlighted the significance of user experience in reducing instrument failure.

Surface defects including machining scratches and grooves, which are termed as stress concentrators, tend to increase the microcrack initiation and fracture life. Kuhn *et al.* [26] reviewed fracture life with the help of SEM and found that surface finish of NiTi files was not always satisfactory. The majority of files showed two to seven or more surface defects in most of the files, which reflect the insufficient manufacturing and packaging processes. These defects could reduce the instrument's integrity and life in clinical applications. In this study, Mtwo files revealed the most severe pitting at all intervals of five, ten and fifteen uses. Although deformation without fracture was highly prevalent in these files, cyclic fatigue accounted for failure in the majority of cases at 71.58%. Therefore, keeping within the stated usage limits by the manufacturer and regular instrument replacement are essential to prevent unanticipated failure [27]. Stress concentrators, such as surface grooves because of the machining process, amplify local stress, which encourages the initiation of microcracks and lower the instrument life [28]. No system tested was flexible enough to achieve superiority in flexibility and uniform load distribution for both bending and torsion. Nevertheless, Twisted File demonstrated superior resistance to surface damage, with the minimum pit arraying compared with other systems. Mtwo files showed the maximum susceptibility towards surface damage. This should be mentioned in light of the fact that, even though this study tried to simulate the geometry and operational conditions of NiTi instruments closely, the stress patterns in the active canal preparation may be different when the instrument comes into contact with the dentinal walls. It highlights the importance of further studies to establish a relationship between instrument design, stress distribution, fatigue fracture and the influence of surface irregularities [29].

The results indicate a necessity of understanding the structural properties and limitations of NiTi rotary systems. Clinicians, while adhering to general rules of rotary instrumentation, need to be cognizant of specific characteristics that might affect the performance and potential for fracture of an instrument. Only high utilization, in the form of hands-on training under supervision, in the endodontic educators' training curriculums can provide better safety as well as minimize the chances of failure by developing the required skills to be optimally handled by the practitioner. The investigation had some limitations that must be taken into consideration. First, the sample size of the groups might have been limited, limiting the generalizability of the results. A larger sample size would have contributed to greater statistical power and more robust conclusions. Second, the investigation was performed under controlled *in vitro* conditions, which were not precisely akin to the complexities of a clinical environment. Patient anatomy, operator variation and intraoral conditions, such as temperature, humidity and debris presence, could have affected the performance and wear of the instruments in an actual clinical environment. Third, the investigation tested only surface pitting as an indicator of instrument degradation, whereas other parameters such as cutting efficiency or fracture resistance were not tested. Addition of these other measures would have given a clearer picture of instrument performance. Finally, the uniform number of uses and the identical application of forces may not have represented the heterogeneity encountered in clinical procedures and could affect the external validity of the results. These limitations indicate that more research would be needed to confirm these results in more representative clinical settings.

## Conclusion:

The observed assessments showed that the Twisted File, Group III, had the highest resistance to surface degradation, where the least number of pits were found after repeated use, while the Mtwo file, Group IV, was the most susceptible to surface changes and showed the highest number of pits in all the usage intervals. The Twisted File had a far better surface integrity when compared for the duration of extensive usage, when compared with that of the ProTaper Next (Group I), V Taper 2H (Group II) and Mtwo (Group IV). With regard to the surface quality, the groups ranked in the following order: Twisted File (Group III) > ProTaper Next (Group I) > V Taper 2H (Group II) > Mtwo (Group IV). These findings result in a stark indication that choosing NiTi instruments with optimal durability avoids minimal surface wear and maximizes clinical efficiency.

## Figures and Tables

**Table 1:** Two comparisons that demonstrate the development of pitting on the instrument surface over the course of five usages

**Group Comparisons**	**Group I Mean Diff.**	**p-value**	**Group II Mean Diff.**	**p-value**	**Group III Mean Diff.**	**p-value**	**Group IV Mean Diff.**	**p-value**
Group I	-	-	-2.55	<0.001	-4.85	<0.001	-5.7	<0.001
Group II			-	-	-3.6	<0.001	-2.3	<0.001
Group III					-	-	-5.9	<0.001

**Table 2:** Two comparisons of the instrument surface's pitting progress during 15 consecutive usages (*p-values are computed using the Post-Hoc Tukey HSD test)

**Group Comparisons**	**Group I Mean Diff.**	**p-value**	**Group II Mean Diff.**	**p-value**	**Group III Mean Diff.**	**p-value**	**Group IV Mean Diff.**	**p-value**
Group I	-	-	-2.35	<0.001	4.9	<0.001	-5.95	<0.001
Group II			-	-	7.25	<0.001	-3.6	<0.001
Group III					-	-	-10.85	<0.001

**Table 3:** Combined table of intragroup comparisons across usage intervals

**Pair Comparison**	**n**	**Mean**	**SD**	**Mean Difference**	**t-value**	**p-value**
Files After 5 Uses	10	7.35 ± 0.85	0.85	-5.7	-15.88	<0.001
Files After 10 Uses	10	13.05 ± 0.86	0.86	-4.75	-18.21	<0.001
Files After 15 Uses	10	17.80 ± 0.89	0.89	-10.45	-27.26	<0.001
